# Prevalence and predictors of scar contracture-associated re-hospitalisation among burn inpatients in China

**DOI:** 10.1038/s41598-021-94432-w

**Published:** 2021-07-22

**Authors:** Zhe Zhu, Weishi Kong, Haibo Wang, Yongqiang Xiao, Ying Shi, Lanxia Gan, Yu Sun, Hongtai Tang, Zhaofan Xia

**Affiliations:** 1 Department of Burn Surgery, the First Affiliated Hospital of Naval Medical University, Burn Institute of PLA, Research Unit of Key Techniques for Treatment of Burns and Combined Burns and Trauma Injury, Chinese Academy of Medical Sciences, Shanghai, 200433 China; 2 Clinical Trial Unit, The First Affiliated Hospital of Sun Yat-Sen University, Guangzhou, 510080 China; 3 Centre for Data Science in Health and Medicine, Peking University, Beijing, 100191 China; 4 Department of Burn and Plastic Surgery, The 970Th Hospital of People’s Liberation Army, Yantai, 264000 Shandong China; 5 China Standard Medical Information Research Center, 288 Haide 2nd road, Shenzhen, 518000 China

**Keywords:** Skin diseases, Trauma, Risk factors

## Abstract

Scar contracture, a common destructive complication causing increased re-hospitalisation rate of burn survivors and aggravated burden on the medical system, may be more seriously in Chinese population because of their higher susceptibility to scar formation. This study aims to evaluate the prevalence and predictors of scar contracture-associated re-hospitalisation among Chinese burn inpatients. This cross-sectional study screened burn inpatients hospitalised during 2013 to 2018 through the Hospital Quality Monitoring System database, among whom re-hospitalised for scar contracture were identified. Variables including sex, age, occupations, burn area, burn site and surgical treatment were analysed. Potential predictors of scar contracture-associated re-hospitalisation among burn inpatients were determined by univariate regression analyses. Of the 220,642 burn inpatients, 2146 (0.97%) were re-hospitalised for scar contracture. The re-hospitalised inpatients were predominantly men and blue-collar workers, showing younger median age at the time of burns, larger burn sizes, and higher percentage of surgical treatment compared other burn inpatients. Significant univariate predictors of scar contracture-associated re-hospitalisation included male sex, age < 50 years, blue-collar work, ≥ 40% total body superficial area burned, inhalation injured, and surgical treatment. Scar contracture is an intractable complication and a significant factor to increase re-hospitalisation rate among Chinese burn inpatients.

## Introduction

Scar contracture, one of the most common post-burn injury complications, attracts attention from clinicians due to its high morbidity rate^1^, crucial impacts on burn survivors’ quality of life, and aggravating burden on the public health system^2^. It commonly occurs in areas around joints and fine motor parts, including the dorsum of the hands or feet and facial area (e.g., eyelid ectropion, microstomia, and nasolabial contracture)^3^. Most researchers measured the range of motion (ROM) of affected joints to evaluate the degree of function limitation caused by scar contracture^3–5^. In addition to the limited ROM, the number of contractures, feelings of traction or restraint in varying degrees, and physical function or aesthetic damage of affected body parts also contribute greatly to its adverse impact^6–8^. Individual differences complicate the establishment of a robust method to assess scar contracture severity.

Besides scar contracture’s physical impact on burn patients, patients’ attitude towards the disease may be influenced by factors including their demand for physical rehabilitation, basic health status, self-efficacy, social support, financial stress, and local medical service level. However, the clinical management status and consumed medical resources of post-burn scar contracture remain unknown, especially in China, the largest developing country, with a population predisposed to scar development^9–11^.

In this study, we screened hospitalised burn patients from 2013 to 2018 using a Chinese nationwide medical database and explored the scar contracture-associated re-hospitalisation rate among burn inpatients. Re-hospitalised inpatients were considered to suffer severe scar contracture and exhaust limited medical resources. Their demographic and clinical characteristics were analysed to understand domestic clinical treatment status and determine predictors of re-hospitalisation for scar contracture after burns, which is meaningful for emphasizing early medical intervention to inhibit scar formation and optimising the allocation of limited hospitalisation medical resources.

## Materials and methods

### Data source

Medical records of burn inpatients discharged between January 1, 2013, and December 31, 2018, were retrospectively extracted from the Hospital Quality Monitoring System (HQMS) database^12^. The HQMS was established in 2013 with the approval of the Bureau of Medical Administration of the Peoples Republic of China to collect hospitalisation information, summarised in a standardized electronic record^13^, and monitor healthcare status in China. The electronic record comprises 346 data acquisition items used for collecting medical data of each inpatient, such as demographic characteristics, clinical diagnoses, patient's identity information, and treatment strategies. Until August 2019, the database contained medical data submitted from 1064 tertiary hospitals.

### Study design

The elementary unit in the HQMS database is the hospitalised case, namely one person-time hospitalisation. Cases with length of stay ≥ 1 day (less than one day is regarded as one day) are considered valid and analysed in our study. Each case contains up to eleven diagnoses coded according to the International Statistical Classification of Diseases and Related Health Problems, 10th Revision (ICD-10), which is specifically described elsewhere^14^. Among them, the primary diagnosis is unique and of great significance for the patient's admission, representing a disease of most harmful to health, most resource-consuming, and longest hospital staying. Those who primarily diagnosed with burns (the ICD-10 codes of burns: T20–T32, X00–X19, and X49.906) were identified as burn cases.

Subsequently, identity information, re-coded in the HQMS database based on the Chinese citizen's identification card number to protect the privacy of each patient, was used to identify burn inpatients. It means that an identified inpatient corresponds to more than one hospitalised cases. Considering that a person was less likely to suffer another severe burn during this study’s observation period, the earliest burn case was determined as the index hospitalisation for burn inpatients.

Thereafter, scar contracture-hospitalised cases were screened by their primary diagnosis code (the ICD-10 codes of scar contracture: H02.002, H02.102, H02.804, H02.807, H02.809, H05.300, H61.101, K13.706, L90.502, M20–M21, M24.208, M24.5, M62.4, M67.0 – M67.1, M72.0, M95) and scar contracture inpatients were identified through the identity information as above mentioned.

In conclusion, inpatients satisfying one of the following exclusion criteria were excluded: (1) discharged outside the period 2013.01.01 to 2018.12.31; (2) a primary diagnosis inconsistent with ICD-10 codes for burns or scar contracture; (3) missing/unknown patient's identity information; (4) repeat submitted records.

Finally, matched by the identity information, burn inpatients eventually re-hospitalised for scar contracture (hereafter referred to as re-hospitalised inpatients) were selected. The exclusion criteria were as follows: 1) not included in the groups of burn inpatients and scar contracture inpatients simultaneously; 2) hospitalisation for scar contracture preceded that for burns.

**Figure 1:**
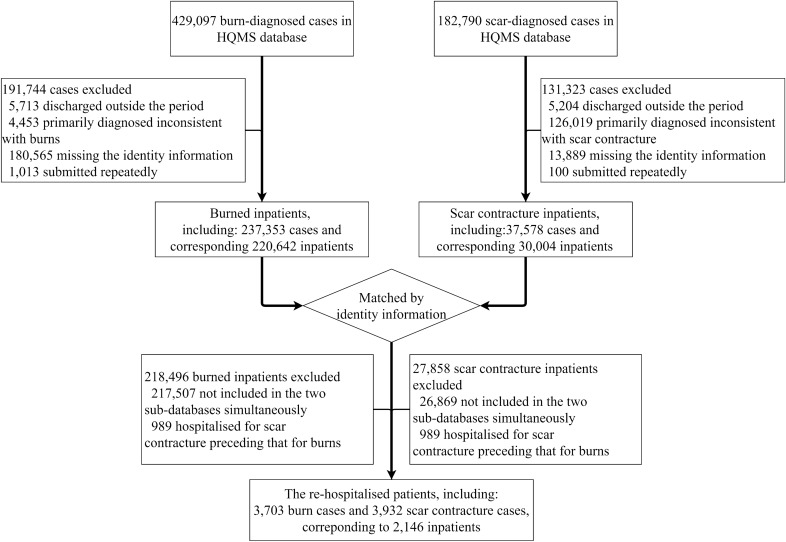
Flow chart for identifying burn inpatients and scar contracture-associated re-hospitalised inpatients. Abbreviation: *HQMS* Hospital Quality Monitoring System.

This study was approved by the *Shanghai Changhai Hospital Ethics Committee* in China (Approval Number: CHEC2014-096) and informed consent was waived with the *Shanghai Changhai Hospital Ethics Committee*. All methods were performed in accordance with the relevant guidelines and regulations. A flow chart highlighting the study design details is shown in Fig. 1.

### Evaluation of variables

The variables in this study including demographic characteristics, such as sex, age at the time of burn injury, and patient’s occupation, and clinical data, such as the burn area, the site of burns, and surgical treatment strategies, are routinely available in the HQMS database. The incidence of scar contracture-associated re-hospitalisation (hereafter referred to as IOC) among burn inpatients, namely the ratio of re-hospitalised inpatients to burn inpatients, was stratified according to these variables. Those with variable(s) missing or unknown were temporarily eliminated if the said variable was analysed.

There are specialized collection items for sex, age, occupation, surgical treatment in the standardized electronic record, while the information of the area and site of burns are included in certain diagnostic codes. For instance, patients with a diagnostic code of T31.100, namely, burns involving 10–19% of body surface, were grouped as 10–19% total body superficial area (TBSA) burned. Unfortunately, a considerable data missing/unknown rate was inevitable as not all patients contain such diagnostic codes.

The ICD-10 codes of TBSA burned are grouped as follows: 0.1–9.9%: T31.0, T32.0; 10–19.9%: T31.1, T32.1; 20–29.9%: T31.2, T32.2; 30–39.9%: T31.3, T32.3; 40–49.9%: T31.4, T32.4; 50–59.9%: T31.5, T32.5; 60–69.9%: T31.6, T32.6; 70–79.9%: T31.7, T32.7; 80–89.9%: T31.8, T32.8; 90–100%: T31.9, T32.9.

Similarly, information concerning the site of burns was extracted from the diagnostic codes. Inpatients with more than one site burned were analysed in corresponding groups respectively. It should be noted that the site of burn rather than the site of scar contracture was analysed due to data missing of the latter in the HQMS database. This means that, for instance, an inpatient in the group of lower limbs burned might hospitalise for hand scar contractures. The ICD-10 codes of burn sites are grouped as follows: Cranio-facio-cervical region: T20, T26; Upper limbs: T22, T23; Lower limbs: T24, T25; Trunk: T21 (001-143)/(203-143)/(302-343); Buttocks and perineum: T21 (151-191)/(251-291/(351-391); Inhalation injury: T27; Multiple regions: T29.

The age at the time of the burn injury was obtained directly in the HQMS database. Although there is no consensus on age-grouping methods, international and domestic experts in the field of burn injuries generally define children as persons aged < 16^15,16^. Children and adults in this study were divided into twelve age groups according to the guidelines in the United States *2016 National Burn Repository Annual Report*. The occupations of patients were classified as blue-collar workers, office staffs, the self-employed and freelance, the unemployed, farmers, retirees, students, and others.

Additionally, surgical treatment was coded according to the International Classification of Diseases Clinical Modification of 9th Revision Operations and procedures (ICD-9-CM-3). Considering many burn inpatients are hospitalised more than once for burns, only those with no ICD-9-CM-3 codes in each hospitalisation record for burns were assigned to the non-operating group, otherwise the others were assigned to the operating group.

### Statistical analysis

SAS version 9.4 M3 for Windows was used to dispose the database and identify target patients. IBM SPSS Statistics version 19 was used for data analysis, and EXCEL version 2016 was used to draw statistical charts and tables. Unordered categorical data are described as numbers and proportions, and χ^2^-tests were used to compare distributions between re-hospitalised inpatients and other burn inpatients. Continuous variables are presented as medians and interquartile ranges, and the Mann–Whitney *U* test was used to analyse ordinal categorical data and continuous variables violating normal distribution. Univariate regression analyses were used to determine potential predictors of scar contracture-associated re-hospitalisation among burn inpatients, and crude odds ratios (ORs) and 95% confidence intervals (CIs) were calculated to statistically understand the strength of the associations between predictors and the primary outcome measure. P < 0.05 was considered statistically significant.

### Ethics approval

Reviewed and approved by Shanghai Changhai Hospital Ethics Committee. Approval number #CHEC2014-096.

## Results

Overall, 220,642 burn inpatients were evaluated, of whom 2146 (0.97%) were re-admitted for scar contracture. The variables of the burn inpatients and re-hospitalised inpatients were statistically described (Table 1).

**Table 1:** Demographic and medical characteristics of the re-hospitalised inpatients and other burn inpatients.

	Re-hospitalised inpatients		Other burn inpatients		P value	IOC (%)
	Value	Missing/Unknown (%)	Value	Missing/Unknown (%)		
Total number, n	2146	–	218,496	–		0.97
Sex, n (%)		0		0	< 0.001^a^	
Male	1551 (72.27)		146,285 (66.95)			1.05
Female	595 (27.73)		72,211 (33.05)			0.82
Age (year), Median (Quartile 1, Quartile 3)	31 (10.44)	0.09	40 (22.53)	0.15	< 0.001^b^	
Occupation, n (%)		10.90		9.08	< 0.001^a^	
Blue-collar worker	534 (27.93)		34,926 (17.58)			1.51
Office staff	104 (5.44)		9688 (4.88)			1.06
Self-employed and freelance	43 (2.25)		7306 (3.68)			0.59
Unemployed	201 (10.51)		18,766 (9.45)			1.06
Farmer	262 (13.7)		41,109 (20.69)			0.63
Retiree	17 (0.89)		9157 (4.61)			0.19
Student	118 (6.17)		9176 (4.62)			1.27
Others	633 (33.11)		68,531 (34.5)			
TBSA (%), Median (Quartile 1, Quartile 3)	27 (10, 52)	36.39	8 (4.17)	57.97	< 0.001^b^	
Site of burns, n (%)		58.99		43.47		
Cranio-facio-cervical region	520 (59.09)		52,965 (43.31)		< 0.001^a^	0.97
Upper limbs	673 (76.48)		48,063 (39.3)		< 0.001^a^	1.38
Lower limbs	381 (43.30)		55,067 (45.03)		0.30	0.69
Trunk	287 (32.61)		22,257 (18.2)		< 0.001^a^	1.27
Buttocks and perineum	58 (6.59)		3501 (2.86)		< 0.001^a^	1.63
Inhalation injury	449 (51.02)		9437 (7.72)		< 0.001^a^	4.54
Multiple regions	13 (1.48)		1450 (1.19)		0.43	0.89
Surgical treatment strategies, n (%)		–		–	< 0.001^a^	
Operating group	1508 (70.27)		64,417 (29.48)			2.29
Non-operating group	638 (29.73)		154,079 (70.52)			0.41

The denominator of percentage represented in parentheses of “Value” columns is equal to the total number of re-hospitalised inpatients or other burn inpatients subtracting the number of data missing or unknown of each variable, and the numerator is the number of each variable represented in the same cell. χ^2^–tests and Mann–Whitney *U* test were performed to test the difference of distributions between the re-hospitalised inpatients and other burn inpatients regard to those variables and labeled as “a” and “b” in the column of P value, respectively. *IOC* incidence of scar contracture-associated re-hospitalisation. *TBSA* total body superficial area.

### Statistical description of burn inpatients

**Figure 2:**
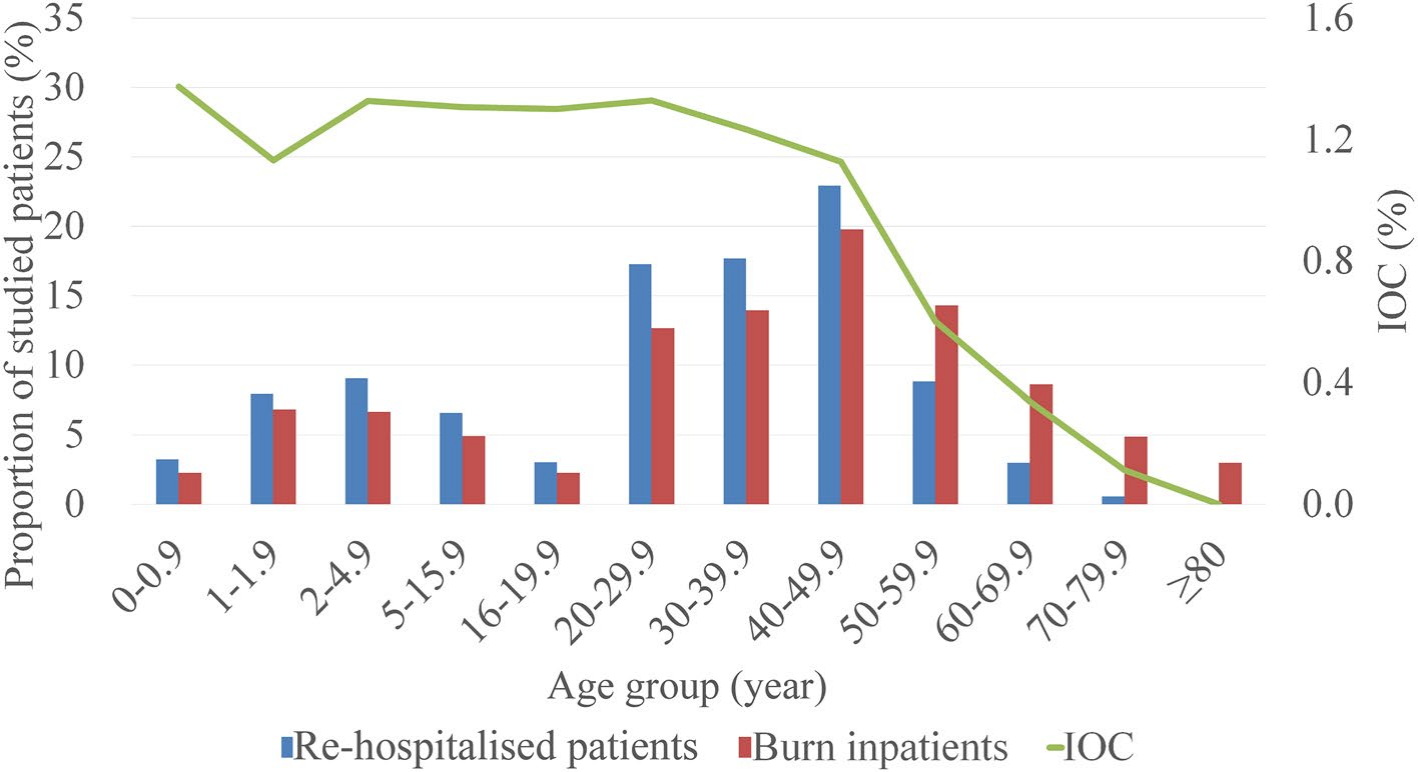
Proportions of burn inpatients and scar contracture-associated re-hospitalised inpatients along with IOC in age groups. The X-axis represents to the age groups classified according to *the 2016 United States National Burn Repository*. The left Y-axis corresponding to the bars represents the proportion of each group in the studied population. The right Y-axis corresponding to the line graph represents the IOC values. Abbreviation: *IOC* the incidence of scar contracture associated re-hospitalisation.

**Figure 3:**
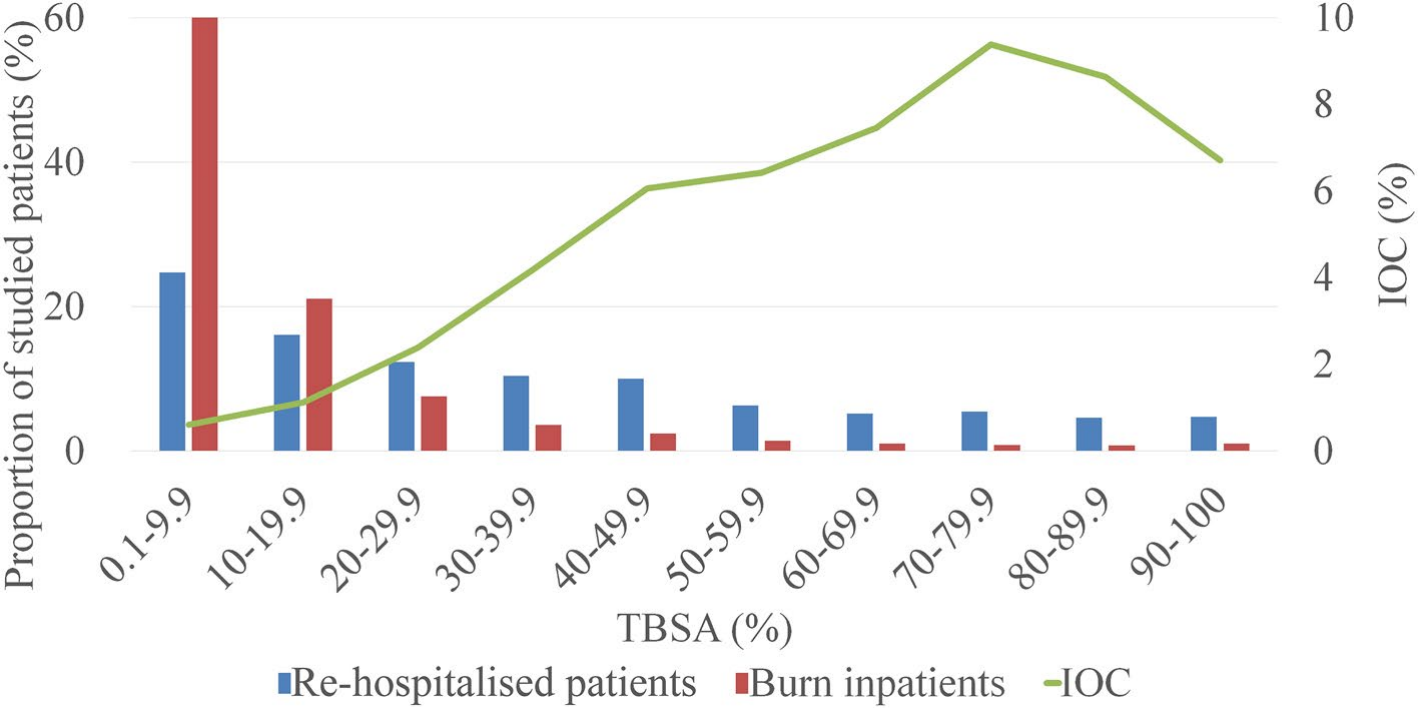
Proportions of burned inpatients and scar contracture-associated re-hospitalised inpatients along with IOC in different TBSA burned groups. The X-axis represents the percentage of TBSA burned groups. The left Y-axis corresponding to the bars represents the proportion of each group in the studied population. The right Y-axis corresponding to the line graph represents the IOC values. Abbreviations: *IOC* the incidence of scar contracture associated re-hospitalisation. *TBSA* total body superficial area.

**Figure 4:**
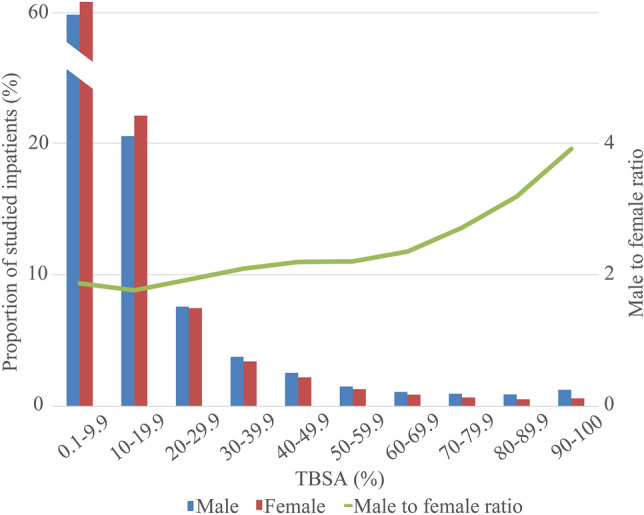
Proportions of burned male and female inpatients along with the male to female ratio in different TBSA burned groups. The X-axis represents the percentage of TBSA burned groups. The left Y-axis corresponding to the bars represents the proportion of each group in the studied population. The right Y-axis corresponding to the line graph represents the values of male to female ratio. Abbreviations: *IOC* the incidence of scar contracture associated re-hospitalisation. *TBSA* total body superficial area.

**Figure 5:**
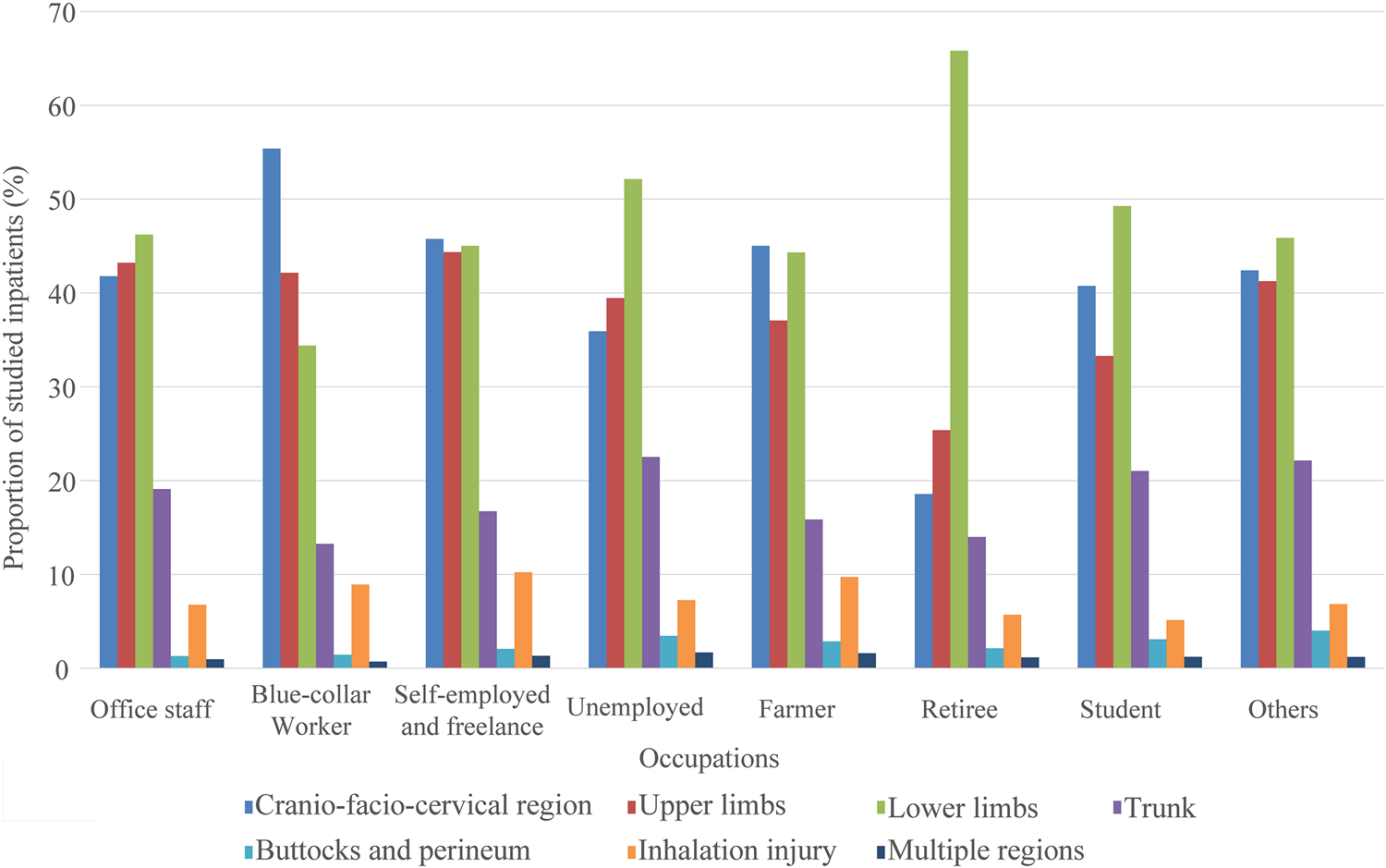
Proportions of different anatomic site burned inpatients in different occupations. The X-axis represents the occupations. The left Y-axis corresponding to the bars represents the proportion of each burn site group in burn inpatients.

Among burn inpatients, 147,836 (67.00%) were males. Excluding 330 inpatients without age specified, there was a bimodal distribution between age groups, mainly in the 1–15 (18.36%) and 20–59 (60.66%) age ranges (Fig. 2). Excluding 20,071 inpatients without occupation specified, burn inpatients were mainly farmers (20.63%) and blue-collar workers (17.68%). Excluding 127,444 inpatients without burn area specified, 60.18% of the remaining had ˂ 10% TBSA burned. As the burn area increased, the number of burn inpatients decreased significantly and those with ≥ 40% TBSA burned constituted 7.56% of patients (Fig. 3), while the male to female ratio increased gradually and the proportion of ≥ 40%TBSA burned was larger in males (8.27% VS 6.21%) (Fig. 4). Excluding 95,321 inpatients without burn site specified, the lower limbs were the most common burn site, followed by the cranio-facio-cervical region and upper limbs (45.02%, 43.31% and 39.57%, respectively). Additionally, the incidence of lower limbs burned was dramatically higher than incidences of other sites among retirees (Fig. 5). Furthermore, 70.12% of patients did not receive surgical treatment.

### Statistical description of re-hospitalised inpatients

The male preponderance is more obvious in re-hospitalised inpatients than in other burn inpatients (72.27% vs. 66.95%, P < 0.001). Excluding two inpatients without age specified, the predominant age ranges of re-hospitalised inpatients were 1–15 (23.56%) and 20–50 (57.84%) (Fig. 2). In addition, the median age of re-hospitalised inpatients was less than that of burn inpatients (31 vs. 40 years, P < 0.001). Excluding 234 inpatients without occupation specified, re-hospitalised inpatients were predominantly blue-collar workers (27.93%) and farmers (13.7%). Excluding 781 inpatients without burn area specified, re-hospitalised inpatients suffered larger burn areas than other burn inpatients (27%TBSA vs. 8%TBSA, P < 0.001). Re-hospitalised inpatients with ˂ 10% TBSA burned constituted 24.76% of the remaining, while those with ≥ 40% TBSA burned constituted a considerable proportion, namely 36.41% (Fig. 3). Among 880 re-hospitalised inpatients with specified burn site, the upper limbs and cranio-facio-cervical region were the top two common burned site. Interestingly, more than half of these inpatients suffered inhalation injuries. Moreover, a higher proportion of re-hospitalised inpatients received surgical treatment for burns than that of other burn inpatients (70.27% vs. 29.48%, P < 0.001).

### Fluctuations in IOC

**Figure 6:**
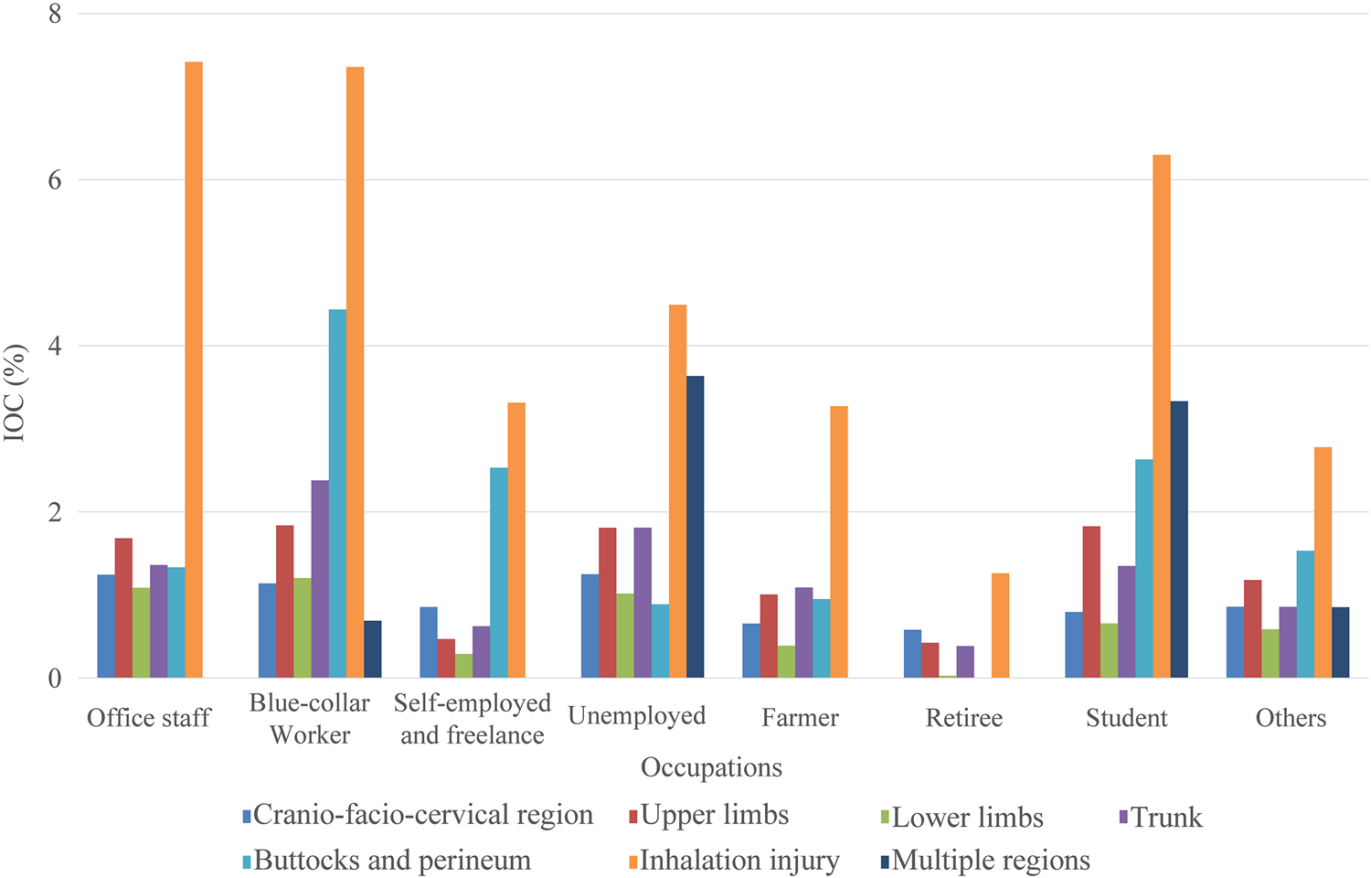
IOC of different anatomic site burned inpatients in different occupations. The X-axis represents the occupations. The left Y-axis corresponding to the bars represents the IOC of each burn site group in burn inpatients. Abbreviation: *IOC* the incidence of scar contracture associated re-hospitalisation.

Overall, 0.97% of burn inpatients were re-hospitalised for scar contracture. IOC among male burn inpatients was higher than that among females (1.05% vs. 0.82%). IOC fluctuated from 1.13 to 1.37% among groups aged < 50 and tended to decline among groups aged ≥ 50 (Fig. 2). IOC of burn inpatients varied among occupations, with blue-collar workers having the highest value (1.51%), followed by students (1.27%). As the burn area became more extensive, IOC increased; however, IOC stably declined among groups with burn areas ≥ 80% TBSA (Fig. 3). Burn inpatients with inhalation injury showed the highest IOC (4.54%), even in different occupations (Fig. 6). The IOCs of inpatients with perineum or hips burned were remarkably higher in blue-collar workers, the self-employed and freelance and students. IOC was higher in the operating group (2.29%) than in the non-operating group (0.41%).

### Predictors assessed by univariate regression analysis

Variables shown in Table 2 were separately analysed by univariate regression analysis. However, the multivariate analysis was incomplete considering the varying degrees of missing/unknown data for the variables. Despite the remarkable influence of confounding factors, crude ORs and 95% CI helped determine potential predictors of scar contracture-associated re-hospitalisation for burn inpatients. Males were 1.29-times more likely to undergo re-hospitalisation for scar contracture after burns than females (crude OR 1.29 [1.17, 1.42], P < 0.001). With the increase of age, the re-hospitalisation risk showed no statistical differences among patients younger than 50 years (crude OR 0.82 [0.62, 1.09], P = 0.17), while significant decreased among patients ≥ 50 years (crude OR 0.92 [0.91, 0.94], P < 0.001). Compared with the former, patients ≥ 50 years were three times more likely to be re-hospitalised for scar contracture (crude OR 3.17 [2.79, 3.61], P < 0.001). Re-hospitalisation risk for blue-collar workers was higher than other occupations, which showed statistical significance expect for the student. Additionally, inpatients with ≥ 40% TBSA burned were seven times more likely to be re-hospitalised for scar contracture than those with ˂ 40% (crude OR 7.46 [6.66, 8.35], P < 0.001). The re-hospitalisation risk of inpatients with inhalation injury were twelve times higher than non-inhalation injured inpatients (crude OR 12.46 [10.90, 14.24], P < 0.001). Moreover, those who received surgical treatment had almost six times higher re-hospitalisation risk than those who did not (crude OR 5.65 [2.15, 6.21], P < 0.001).

**Table 2:** Univariate regression analyses for scar contracture-associated re-hospitalisation among burn inpatients.

	Independent variable	Partial regression coefficient	SE	P	Crude OR	95% CI
Sex	Male	0.252	0.048	< 0.001	1.29	[1.170, 1.415]
	Female	Reference				
Age	< 50 years	− 0.197	0.144	0.171	0.82	[0.620, 1.089]
	≥ 50 years	− 0.800	0.009	< 0.001	0.92	[0.908, 0.940]
	< 50 years	1.154	0.066	< 0.001	3.17	[2.788, 3.608]
	≥ 50 years	Reference				
Occupation	Office staff	− 0.354	0.108	0.001	0.70	[0.568, 0.867]
	Self-employed and freelance	− 0.955	0.159	< 0.001	0.38	[0.282, 0.526]
	Unemployed	− 0.356	0.083	< 0.001	0.70	[0.595, 0.825]
	Farmer	− 0.875	0.076	< 0.001	0.42	[0.359, 0.484]
	Retiree	− 2.108	0.247	< 0.001	0.12	[0.075, 0.197]
	Student	− 0.173	0.102	0.091	0.84	[0.688, 1.028]
	Others	− 0.504	0.059	< 0.001	0.60	[0.538, 0.678]
	Blue-collar worker	Reference				
TBSA burned	40–100%	2.009	0.058	< 0.001	7.46	[6.659, 8.349]
	0–39.9%	Reference				
Site of burns	Inhalation injury	2.522	0.068	< 0.001	12.46	[10.898, 14.243]
	Without inhalation injury	Reference				
Treatment strategies	Surgical treatment (%)	1.732	0.047	< 0.001	5.65	[5.151, 6.205]
	Without operations	Reference				

*OR* odds ratio, *CI* confidence interval.

## Discussion

In this study, we evaluated the rate and associated risk factors of re-hospitalisation caused by post-burn scar contracture in a Chinese population. We found that male sex, age < 50, blue-collar work, ≥ 40% TBSA burned, and surgical treatment were significant predictors.

We identified burn patients hospitalised in 1064 tertiary hospitals; the rate of re-hospitalisation for scar contracture was 0.97%, quite a low rate beyond authors’ expectation when considering that approximately 33–54% of burn inpatients have been reported to suffer from scar contracture at discharge^3,6,17^. It seems to imply a dramatic discrepancy between high morbidity and low re-hospitalisation rate of post-burn scar contracture, even though there is no comparability between our manuscript and those previous studies. On the one hand, the relief of severity and morbidity of scar contracture may be obtained after a long period after burns^17^ or by physical and occupational therapies at the well-established outpatient services^3,18^. On the other hand, factors including patients' physical and mental health, economic conditions, tolerance of current life status, family members’ attitude, and social relations^19^, may significantly influence patients' decisions toward further therapies. In general, hospitalisation is an evitable consequence for burn inpatient with scar contracture, which highlights the significance and importance of our study to evaluate the prevalence and predictors of scar contracture-associated re-hospitalisation among Chinese burn inpatients.

In the present study, univariate analyses indicated that predictors of re-hospitalisation for scar contracture among burn inpatients included male sex, ≥ 40% TBSA burned, and surgical treatment for burns. Similarly, Schneider et al.^6^ and Goverman et al.^3^ found that TBSA burned and skin grafting are predictors of the occurrence, case frequency, and severity of scar contracture in burn inpatients, whereas female sex was a protective factor. The underlying mechanism of sex in the pathogenesis of post-burn scar contracture remains controversial and association between sex and nature of burns needs to be considered. Jeffrey et al.^20^ reported that males suffered more proportions of flame, chemical and electricity/radiation, less proportions of scald/hot object, and larger burn extent of second degree than females. Scholars from Vietnam found that males possessed higher proportions of burn due to dry heat (flame or hot surface contact) and electricity and lower proportion of burn due to scald in 641 adolescent patients^21^ and in 5061 adult patients^22^. The latter also reported larger burn extent in males. Similar in the study by Felicia et al.^23^, there are higher proportion of flame burns, lower proportion of scald burns, and larger percentage of TBSA burned in males than in females. Outcomes in our study also indicated that patients in groups with 20% and above TBSA burned accounted for higher proportions in males than in females. Therefore, further research should take the nature of injury, as well as educational background, income level, and sociocultural environment into consideration to better understand the role of sex in the pathogenesis of scar contracture.

An interesting finding that IOC continuously declined among ≥ 80% TBSA burned groups suggested that the re-hospitalisation rate is weakened by the risk of higher mortality associated with extent of burn area (i.e., ≥ 80% TBSA burned in the present study), despite the positive correlation between TBSA burned and scar contracture.

Moreover, surgical treatment is a crucial method of wound healing^24^, especially for severe burns such as larger burn area, deep partial or full thickness burns, functional regions involved, and wound infected, which are closely associated with hypertrophic scar formation and scar contractures and contribute to the higher re-hospitalisation risk of post-burn scar contracture among surgically treated patients. Clifford et al.^2^ found that the number of operations during burn hospitalisation is a significant predictor of reconstructive procedures within 24 months after burns. Gangemi et al.^25^ considered the number of surgical procedures as an independent predictor of post-burn contractures (OR 1.39 [1.06, 1.82]). The role of surgeries themselves, however, remains unclear in the pathogenesis of scar contractures. The outcomes in our study suggest that, to a certain extent, deliberate and prudent therapies rather than overly and aimlessly aggressive treatments are recommended for burn patients, which has also been recommended in previous studies by Mark et al. and Moi et al ^18,19^. Further research should take the type, number and timing of surgical interventions into consideration to understand the role of surgeries during post-burn scar formation.

This study also highlights the influence of age at the time of the burn injury on re-hospitalisation risk for scar contracture. We found the IOC curve in the 0–50-year groups was a relatively flat plateau. Univariate analysis indicated that there was no statistical difference in re-hospitalisation risk among patients younger than 50. This emphasizes the important role of anti-scar treatment in both adults and children, despite higher predisposition of the latter to scar contracture formation and aggravation owing to their rapid growth and development^18^. Notably, the IOC curve declined abruptly for age groups above 50 years; re-hospitalisation risks significantly decreased as the age increased among patients ≥ 50. This finding is understandable as inpatients burned at age ≥ 50 may have higher risks of post-burn mortality^15^, higher tolerance for functional and aesthetic impacts of scar contracture, more severe underlying diseases that limit further aggressive treatment, and may be less likely to have scars let alone contractures than younger inpatients^26^. These factors contribute to a lower risk of re-hospitalisation for scar contracture.

Occupation is another crucial factor that influences the prognoses of burn patients. Whereas occupational burns are mainly caused by flame/fire and scalds, a considerable proportion of electrical and chemical injuries^27,28^ tend to be more serious and prevalent in males, potentially causing mental disturbances with a serious impact on working ability, thus hindering the easy integration of patients into society^29,30^. We found that the highest proportion of re-hospitalised inpatients were blue-collar workers, who had significantly higher re-hospitalisation risks for scar contracture than other occupations (excluding the student), suggesting that blue-collar workers are subjected to occupational risks related to post-burn scar contracture development and need a functional and established legal system to prevent occupational disabilities.

Inhalation injury plays a crucial role in the adverse outcomes for burn patients during hospitalisation, predicting higher mortality, longer hospital stay, and more complications^31–33^. Currently, the association between inhalation injury and long-term outcomes has gotten more attention and remains unclear. A retrospective study involving 1871 burn inpatients indicated that inhalation injury was a significant predictor for unemployment at 24 months after burns, while was not statistically associated with health-related quality of life outcomes^34^. Additionally, in a recent study by Cordelie et al.^35^, inhalation injury showed non-significant association with post-discharge mortality and all-cause readmission. In our study, however, the IOC was dramatically high among inpatients with inhalation injury, which turned to be a univariate predictor of re-hospitalisation for scar contracture. It is better to take the potential links between inhalation injury and deeper and larger burns into consideration to understand these findings. Overall, further well-designed research is need to investigate the long-term impacts of inhalation injury in burn patients.

This study had limitations. Firstly, re-hospitalisation incidence for scar contracture may be underestimated as some burn inpatients were re-hospitalised after 2018, others admitted for burns before 2013, and some target patients admitted to hospitals without submitting data to the HQMS database. This defect may improve with the increasing HQMS-database coverage and longer observation periods. Secondly, the standardized electronic record is designed for all admitted patients and monitoring hospital quality. Correspondingly, the HQMS database has limitations in analysing specific conditions, including burns or scar contracture, as essential factors like the site and number of scar contractures, the aetiology and depth of burns, and detailed non-operative and operative treatment strategies are beyond the data collection range. However, this drawback greatly motivates the establishment of specialized medical databases for burn patients. Finally, we must acknowledge the inevitable selection bias owing to the focus on patients discharged from tertiary hospitals, which utilize relatively more rigorous procedures. However, it should be noted that tertiary hospitals in China possess well-developed outpatient health services and provide fundamental and specialty medical services for patients nationwide. This helped ensure that the inpatients we studied truly required hospitalisation and were representative of patients countrywide.

## Conclusion

Scar contracture is a significant cause for re-hospitalisation in Chinese burn inpatients. The re-hospitalised inpatients were younger and suffered larger burns than other burn inpatients. The potential predictors of re-hospitalisation for scar contracture among burn inpatients are male sex, age < 50, blue-collar work, ≥ 40% TBSA burned, with inhalation injury, and surgical treatment.

## Abbreviations

CI: Confidence interval
HQMS: Hospital Quality Monitoring System
ICD-10: International Statistical Classification of Diseases and Related Health Problems, 10th Revision
IOC: Incidence of scar contracture-associated re-hospitalisation
OR: Odds ratio
TBSA: Total body superficial area
